# Supplementary material for: Flooding and elevated prenatal depression in rural Bangladesh: A mixed methods study
Source: PLOS Glob Public Health. 2025 Jul 21;5(7):e0004792. doi: 10.1371/journal.pgph.0004792 (PMC12279153; doi:10.1371/journal.pgph.0004792)
Supplement: S1 Table — (DOCX) [file pgph.0004792.s001.docx]

##### **S1 Table: Characteristics and flood experiences of participants**

| **Characteristic** | | **N exposed** | **Mean/% exposed** | | **N unexposed** | | | **Mean/% unexposed** |
| --- | --- | --- | --- | --- | --- | --- | --- | --- |
| **Participant characteristics** | |  |  | |  | | |  |
| Mother's years of education | | 93 | 5.6 | | 785 | | | 6.3 |
| Spouse’s years of education | | 93 | 5.1 | | 785 | | | 5.2 |
| Mother's age in years | | 93 | 24.9 | | 785 | | | 24.4 |
| Gestational age in weeks | | 93 | 24 | | 785 | | | 23.4 |
| **Household characteristics** | |  |  | |  | | |  |
| Number of household members | | 93 | 5.1 | | 785 | | | 4.9 |
| Roof material is bamboo | | 93 | 0.00% | | 785 | | | 0.10% |
| Roof material is tin | | 93 | 100.00% | | 785 | | | 99.90% |
| Wall material is jute, bamboo, or straw | | 93 | 5.40% | | 785 | | | 3.30% |
| Wall material is brick or cement | | 93 | 0.00% | | 785 | | | 0.60% |
| Wall material is tin | | 93 | 94.60% | | 785 | | | 96.10% |
| Floor material is mud | | 93 | 100.00% | | 785 | | | 100.00% |
| **Sanitation*** | |  |  | |  | | |  |
| Basic | | 92 | 14.10% | | 785 | | | 17.60% |
| Limited | | 92 | 8.70% | | 785 | | | 12.40% |
| Unimproved | | 92 | 77.20% | | 785 | | | 70.10% |
| Open defecation of children <3 years | | 93 | 25.80% | | 785 | | | 20.30% |
| Handwashing station with soap and water | | 93 | 24.70% | | 785 | | | 23.20% |
| Basic water* | | 93 | 100.00% | | 785 | | | 100.00% |
| **Sociodemographic characteristics** | |  |  | |  | | |  |
| Monthly income < 12,001 taka | | 93 | 64.50% | | 785 | | | 58.60% |
| Has electricity | | 93 | 93.50% | | 785 | | | 97.80% |
| Owns mobile | | 93 | 98.90% | | 785 | | | 97.70% |
| Owns livestock | | 93 | 84.90% | | 785 | | | 78.50% |
| Father works in agriculture | | 93 | 20.40% | | 785 | | | 19.00% |
|  | | | | | | | | |
| **Flooding** | | **N** | | | **Mean/ %** | | | |
| Union flooded for at least one day in past 6 months | | 878 | | | 10.3% | | | |
| Compound flooded for at least one day in past 6 months | | 881 | | | 3.6% | | | |
| Number of months ago that the compound flooded | | 32 | | | 2.3 | | | |
| Inside of the home flooded in the past 6 months | | 881 | | | 0.30% | | | |
| Number of days the home was flooded | | 3 | | | 14 | | | |
| Latrine flooded in past 6 months | | 881 | | | 1.2% | | | |
| Number of days latrine was flooded | | 11 | | | 12.5 | | | |
| Tubewell flooded in past 6 months | | 881 | | | 0.6% | | | |
| Number of days the tubewell was flooded | | 5 | | | 11.4 | | | |
| Respondent feels prepared to handle a flood if it happened tomorrow | | 879 | | | 28.6% | | | |
| **Characteristic** | **N exposed** | **Mean/% exposed** | | **N unexposed** | | **Mean/% unexposed** |  |  |
| **Participant characteristics** |  |  | |  | |  |  |  |
| Mother's years of education | 93 | 5.6 | | 785 | | 6.3 |  |  |
| Spouse’s years of education | 93 | 5.1 | | 785 | | 5.2 |  |  |
| Mother's age in years | 93 | 24.9 | | 785 | | 24.4 |  |  |
| Gestational age in weeks | 93 | 24 | | 785 | | 23.4 |  |  |
| **Household characteristics** |  |  | |  | |  |  |  |
| Number of household members | 93 | 5.1 | | 785 | | 4.9 |  |  |
| Roof material is bamboo | 93 | 0.00% | | 785 | | 0.10% |  |  |
| Roof material is tin | 93 | 100.00% | | 785 | | 99.90% |  |  |
| Wall material is jute, bamboo, or straw | 93 | 5.40% | | 785 | | 3.30% |  |  |
| Wall material is brick or cement | 93 | 0.00% | | 785 | | 0.60% |  |  |
| Wall material is tin | 93 | 94.60% | | 785 | | 96.10% |  |  |
| Floor material is mud | 93 | 100.00% | | 785 | | 100.00% |  |  |
| **Sanitation*** |  |  | |  | |  |  |  |
| Basic | 92 | 14.10% | | 785 | | 17.60% |  |  |
| Limited | 92 | 8.70% | | 785 | | 12.40% |  |  |
| Unimproved | 92 | 77.20% | | 785 | | 70.10% |  |  |
| Open defecation of children <3 years | 93 | 25.80% | | 785 | | 20.30% |  |  |
| Handwashing station with soap and water | 93 | 24.70% | | 785 | | 23.20% |  |  |
| Basic water* | 93 | 100.00% | | 785 | | 100.00% |  |  |
| **Sociodemographic characteristics** |  |  | |  | |  |  |  |
| Monthly income < 12,001 taka | 93 | 64.50% | | 785 | | 58.60% |  |  |
| Has electricity | 93 | 93.50% | | 785 | | 97.80% |  |  |
| Owns mobile | 93 | 98.90% | | 785 | | 97.70% |  |  |
| Owns livestock | 93 | 84.90% | | 785 | | 78.50% |  |  |
| Father works in agriculture | 93 | 20.40% | | 785 | | 19.00% |  |  |

* Sanitation and water defined using the WHO/JMP water and sanitation ladders.
