# Supplementary material for: Flooding and elevated prenatal depression in rural Bangladesh: A mixed methods study
Source: PLOS Glob Public Health. 2025 Jul 21;5(7):e0004792. doi: 10.1371/journal.pgph.0004792 (PMC12279153; doi:10.1371/journal.pgph.0004792)
Supplement: S2 Table — (DOCX) [file pgph.0004792.s002.docx]

**S2 Table: E-values for prevalence ratios with depression**

| **Outcome** | **E-value** |
| --- | --- |
| **Moderate or severe depression** |  |
| Flooded latrine | 6.61 |
| Flooded compound | 3.58 |
| Flooded union | 2.16 |
| **Severe depression** |  |
| Flooded latrine | 8.13 |
| Flooded compound | 2.19 |
| Flooded union | 1.67 |
