# Supplementary material for: Flooding and elevated prenatal depression in rural Bangladesh: A mixed methods study
Source: PLOS Glob Public Health. 2025 Jul 21;5(7):e0004792. doi: 10.1371/journal.pgph.0004792 (PMC12279153; doi:10.1371/journal.pgph.0004792)
Supplement: S3 Table — (DOCX) [file pgph.0004792.s003.docx]

##### **S3 Table: Association between flooding and EPDS score**

|  | **N** | **Mean among exposed** | **Mean among unexposed** | **Crude mean difference (95% CI)** | **Adjusted * mean difference (95% CI)** |
| --- | --- | --- | --- | --- | --- |
| Flooded latrine | 881 | 9.5 | 5.7 | 3.76 (0.74, 6.78) | 3.87 (0.72, 7.02) |
| Flooded compound | 881 | 8.3 | 5.6 | 2.67 (0.58, 4.75) | 2.34 (0.13, 4.55) |
| Flooded union | 878 | 6.6 | 5.6 | 0.98 (-0.21, 2.17) | 0.86 (-0.30, 2.02) |

*Adjusted models adjusted for any of the following covariates that were associated with each outcome (likelihood ratio p-value<0.2): for month, wealth index, mother’s years of education, spouse’s years of education, mother’s age, gestational age
