# Supplementary material for: Flooding and elevated prenatal depression in rural Bangladesh: A mixed methods study
Source: PLOS Glob Public Health. 2025 Jul 21;5(7):e0004792. doi: 10.1371/journal.pgph.0004792 (PMC12279153; doi:10.1371/journal.pgph.0004792)
Supplement: S4 Table — (DOCX) [file pgph.0004792.s004.docx]

##### **S4 Table: Association between distance to surface water and moderate/severe depression and severe depression**

|  | **N** | **Crude prevalence ratio (95% CI)** | **Adjusted * prevalence ratio (95% CI)** |
| --- | --- | --- | --- |
| **Moderate or severe depression** |  |  |  |
| Seasonal water | 881 | 0.92 (0.34, 2.48) | 1.02 (0.34, 3.05) |
| Permanent water | 881 | 0.92 (0.80, 1.05) | 0.95 (0.82, 1.10) |
| **Severe depression** |  |  |  |
| Seasonal water | 881 | 0.13 (0.01, 1.13) | 0.19 (0.02, 1.78) |
| Permanent water | 881 | 0.79 (0.66, 0.94) | 0.86 (0.71, 1.04) |

*Adjusted for month, wealth index, mother’s years of education, spouse’s years of education, mother’s age, gestational age
