# Supplementary material for: Flooding and elevated prenatal depression in rural Bangladesh: A mixed methods study
Source: PLOS Glob Public Health. 2025 Jul 21;5(7):e0004792. doi: 10.1371/journal.pgph.0004792 (PMC12279153; doi:10.1371/journal.pgph.0004792)
Supplement: S5 Table — (DOCX) [file pgph.0004792.s005.docx]

##### **S5 Table: Association between distance to surface water and EPDS score**

|  | **N** | **Crude mean difference (95% CI)** | **Adjusted * mean difference (95% CI)** |
| --- | --- | --- | --- |
| Seasonal water | 881 | -1.67 (-3.57, 0.22) | -1.42 (-3.40, 0.57) |
| Permanent water | 881 | -0.35 (-0.52, -0.17) | -0.27 (-0.44, -0.10) |

*Adjusted for month, wealth index, mother’s years of education, spouse’s years of education, mother’s age, gestational age
