# Supplementary material for: Flooding and elevated prenatal depression in rural Bangladesh: A mixed methods study
Source: PLOS Glob Public Health. 2025 Jul 21;5(7):e0004792. doi: 10.1371/journal.pgph.0004792 (PMC12279153; doi:10.1371/journal.pgph.0004792)
Supplement: S2 File — (DOCX) [file pgph.0004792.s007.docx]

**Effects of building home concrete floors on child health: an experimental study in a selected area of Bangladesh**

**Climate Resilience FGD Guideline**

**Table of Contents**

[**Annex 1.1:** Guidelines for Focus Group Discussions with ***Adult Female*** 3](#_Toc200629241)


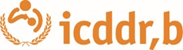


# **Annex 1.1:** Guidelines for Focus Group Discussions with ***Adult Female***

| **Protocol No. PR-22069** | **Version No. 2.00** | **Date: 12-06-24** |
| --- | --- | --- |

**Protocol Title:** Effects of building home concrete floors on child health: an experimental study in a selected area of Bangladesh

**Principal Investigator’s name:** Dr. Md. Mahbubur Rahman

**Organization:** International Centre for Diarrhoeal Diseases Research, Bangladesh (icddr,b)

**Section 1: General information about the participants of the focus group discussion**

| **SL No** | **Name** | **Profession** | **Age** | **Education** |
| --- | --- | --- | --- | --- |
| 1 |  |  |  |  |
| 2 |  |  |  |  |
| 3 |  |  |  |  |
| 4 |  |  |  |  |
| 5 |  |  |  |  |
| 6 |  |  |  |  |
| 7 |  |  |  |  |
| 8 |  |  |  |  |

**Section 2: Experiences with extreme weather**

- The first thing I’d like to discuss are your experiences with extreme weather. Have you ever experienced this? What about extreme flooding of your home [add definition]? How often does this happen? How high was the water inside your home? What happened to you and your family?
- What about other weather events like extreme heat? What happened to you and your family in the May heatwave when the temperatures were over 40C for over a week [add name of heat wave]?
- What materials is your house made of? During floods, have you experienced damage to your house, livestock, tube well, or other assets?
- Did you face any additional challenges due to your gender or age during the flood/heat waves? (Probe: challenges for women, clothing, skills, capabilities) Who are the most vulnerable groups during any extreme weather events and why? (probe: pregnant women, infants and young children, disable people)

**Section 3: Adaptation strategies**

- What were your household’s adaptation strategies to tackle those extreme weather events?
- Do different members of your family have different responsibilities during floods? What were your strategies? What are the basic survival skills (swimming, climbing a tree, boating etc.) that are required for a person to survive during the flood? Are you capable of those skills?
- In case of the worst of the negative events, how long would it take for your household to return to a satisfactory situation? Have you ever added special materials to your house to protect it against floods? Have you ever needed to move your house structure after floods? Were you able to implement those changes, or were there reasons you could not? How long did it take you to implement those changes?

**Section 4: Income Impacts**

- What are the sources of your family’s income? How do floods influence your family’s income? (Probe: daily work, job loss during flood, for how long, dealing with income loss, transportation)
- How about when there is extreme heat? What about if there is extreme drought and rivers dry up? (Probe: influence on income, daily work, transportation)
- How do you think the male figures in your household respond to income stress during floods? How do you personally deal with income stress? Have you even started a new way to have income after your family faced losses because of flooding, extreme heat, or drought?
- Have you ever experienced domestic violence during floods or observed violence? (Probe: By whom, for what, consequence) Is any of your child married? (Probe: when, child’s age, gender)

**Section 5: Displacement/Shelters**

- During floods, if you can’t stay in your home, where have you gone immediately for shelter? How do you decide where to go? Do you feel like you have the chance to voice your opinion in your household about where to go?
- During floods, tell me about how you decide whether to go to shelters, and the conditions within the shelters. Have you been to a shelter? Do women and men have separate areas for sleeping and using the toilet in shelters, and what do you think about that? Do you face any challenges there? Do you think adolescent girls face any unique challenges in the shelters? Do you have access to enough food and clean water, and medical supplies within shelters (including for pregnant women, infants and young children)? What happens if someone has a medical emergency within the shelters?
- Have you observed or experienced gender-based violence/discrimination within shelters? If so, what do you think causes this violence/discrimination? If you could make any changes to the flood shelters, what would you do?

**Section 6:** **Migration**

- Do you think moving (temporarily / permanently) from your home village will be necessary to escape the floods? If so, when? Do you think every individual in your family should move? What factors are encouraging you to move? What factors are preventing you from moving? Where do you think you would go if you moved? Why would you go there?
- Who makes decisions in your household about migration? Do you ever have discussions in your household about migration? Do you feel like you have the chance to voice your opinion about migration in your household?

**Section 7: School Enrollment/Attendance**

- Are children attending school [age range, gender]? What influences school attendance?
- During floods, how long do schools usually close? Have any of your child dropped out from school or faced session jam due to constant crisis of migration/flood/heat wave? Have you ever needed to stop one of your children’s enrollment in school because of income losses during floods? If so, how do you choose which child needs to stop attending school?
- How about when the weather was extremely hot, how long did schools stay closed? What did your children do when not in school when it was extremely hot?

**Section 8: Health**

- During floods, how do you access water? How does the flooding affect what and when you eat? How is your hygiene and sanitation affected? How does the flooding affect care for infants and young children? Do you face any health problems due to floods? How does the flooding affect access to healthcare?
- What about when the weather was extremely hot? (Probe: impact on health, influence on accessing water, food consumption, hygiene and sanitation, infants and young children, access to healthcare)

**Section 9:** **Menstruation during Floods**

- How do you manage menstruation during floods? Do you stockpile any menstrual health materials before floods? If you visit shelters during floods, how do you manage your menstruation there? What emotions do you feel when managing menstrual hygiene during floods?

**Section 10: Anticipatory Behaviors**

- How do you think floods’ frequency and severity will change in the future? What about heat wave frequency and severity? How does thinking about these future predictions of floods and heat waves make you feel? Do you think about these future predictions often?
- Do you think you need to make any changes to your house infrastructure to protect it against future floods? If so, what changes would you want to make? Are these changes expensive/do you think you will need to save money for these changes? Do you ever save food, water, medicine, or other materials for floods? Do you save money for floods? Would you rather save money for floods or directly purchase protective house materials beforehand?
- Do you think you need to make any changes to what you do to prepare for future heat waves? Are these changes expensive/do you think you will need to save money for these changes?
- Does your community prepare for flood events in the future? What physical or other structures might protect against these extreme events? What resources do you think the government/aid organizations should provide to help your community prepare for future floods?


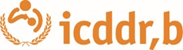


- in your household about migration? Do you feel like you have the chance to voice your opinion about migration in your household?

**Section 7: School Enrollment/Attendance**

- Are you attending school? What influences your school attendance?
- During floods, how long do schools usually close? Have any of you ever dropped out from school or faced session jam due to constant crisis of migration/flood/heat wave? Have you ever needed to stop your enrollment in school because of your family’s income losses during floods? Has any of your siblings also needed to stop their education? What did you do when not in school during floods?
- How about when the weather was extremely hot, how long did schools stay closed? What did you do when not in school when it was extremely hot?

**Section 8: Health**

- During floods, how do you access water? How does the flooding affect what and when you eat? How is your hygiene and sanitation affected? How does the flooding affect care for infants and young children? Do you face any health problems due to floods? How does the flooding affect access to healthcare?
- What about when the weather was extremely hot? (Probe: impact on health, influence on accessing water, food consumption, hygiene and sanitation, infants and young children, access to healthcare)

**Section 9: Anticipatory Behaviors**

- How do you think floods’ frequency and severity will change in the future? What about heat wave frequency and severity? How does thinking about these future predictions of floods and heat waves make you feel? Do you think about these future predictions often?
- Do you think you need to make any changes to your house infrastructure to protect it against future floods? If so, what changes would you want to make? Are these changes expensive/do you think your family will need to save money for these changes? Do your family ever save food, water, medicine, or other materials for floods? What kind of precautions and preparations do you take for floods? Do you save anything for floods?
- Do you think your family need to make any changes to what they do to prepare for future heat waves? Are these changes expensive/do you think your family will need to save money for these changes?
- Does your community prepare for flood events in the future? What physical or other structures might protect against these extreme events? What resources do you think the government/aid organizations should provide to help your community prepare for future floods?
