# Supplementary material for: Flooding and elevated prenatal depression in rural Bangladesh: A mixed methods study
Source: PLOS Glob Public Health. 2025 Jul 21;5(7):e0004792. doi: 10.1371/journal.pgph.0004792 (PMC12279153; doi:10.1371/journal.pgph.0004792)
Supplement: Attachment — Submitted filename: PGPH-D-25-00092.docx [file pgph.0004792.s008.docx]

Referee report on “**Flooding and elevated prenatal depression in a climate-sensitive community in rural Bangladesh: a mixed methods study” (PGPH-D-25-00092)**

The paper aims to examine the associational impact of floods on the mental health outcomes, as measured by depression, amongst pregnant women in rural Bangladesh. In principle, the paper uses a simple linear regression model to examine the likelihood of prenatal depression amongst those women who witnessed a flood incident in the past 6 months in comparison to those who did not. The author(s) report elevated depression levels for the exposed women. The effect are particularly higher if the women experienced latrine flooding.

The paper highlights an important issue relating to prenatal depression and the author(s) efforts are appreciable. I have a few suggestions for the author(s) to consider.

**Major Comments**

1. **Empirical Strategy:**
(a) While authors state that they are using generalized linear model with Gaussian distribution, as per my understanding, this in essence boils down to a simple linear regression model. I urge the author(s) to remove such redundancy and provide more clarity in this context.

Relatedly, author(s) may consider explicitly writing the regression equation in interest of readers.

(b) The empirical strategy compares difference in outcomes of women witnessing a flood event with the women those did not witness such an event in the last 6 months. While flood is a natural disaster (implying it is exogeneous) -- however there are reasons why flooding may an endogenous regressor.

The reason is that the regions (**unions**) who witnessed a flood in last 6 months may actually be more likely to witness a flood in comparison to regions which did not witness a flood. This is evident in Figure 2b and 2c. Households that are nearer to seasonal water streams are more likely to report compound (and, perhaps latrine) flooding.

If the fact that the regions (**unions**) who witnessed a flood in last 6 months may actually be more likely to witness a flood in comparison to regions which did not witness a flood, then there is a strong selection bias. In other words, the affected (treated) mothers are more likely to be facing such floods for a continuously more period of time. Thus there remains a possibility that the estimates which the empirical strategy provides may actually be over estimates.

The reason is that floods may induce a perpetual/continued depression -- that may continue beyond 6 months (which is the survey recall horizon). If such affected women (in regions more likely to witness floods) have been suffering from depression for long, then the estimates provided in the table actually represent combining impact of regular flooding rather than floods in the past 6 months.

Relatedly, there may be confounders such as:

o Pre-existing mental health conditions:

o Women who already suffer from anxiety or depression might perceive and report flooding as more traumatic.

(c) Since authors can potentially know the date when the flood actually occurred (using their data), author(s) can see whether the length of exposure to flood also affects the extent of depression. For instance, are those who suffered a flood just one month prior to the survey more/less likely to be depressed than those who suffered 3 months ago. Related to (b), this may add more insights about the temporal impacts of floods.
(d) · The self-reported flooding data may be prone to recall bias, yet there is no discussion of how this could impact the results.
(e) Clustering of standard errors: Since the outcomes within regions (unions) may be highly correlated, author(s) may want to cluster their standard errors at the regio(union) level.
2. **Introduction Section**

The author(s) may consider adding two additional paragraphs in the Introduction section. One paragraph for adding the key results, and another additional paragraph for adding the highlighting the key contribution of the paper.

3. **Descriptive Statistics**

Author(s) may consider separating the descriptive statistics from the main results. A separate section for descriptive statistics may be added.

Relatedly, Table 1 is a little confusing. While I understand N=32, N=3, N=11 (etc) represents certain subsamples. However, their corresponding numbers in column (2) are not percentages. Author(s) may consider revising the table to avoid any source of confusion.

**Minor Comments**

1. **Table notes**

The author(s) may consider providing detailed table notes describing in detail the outcome variables, the independent variable, and the controls along with a brief about methodology (if deemed necessary). The idea is that the reader should be able to understand the paper just by glancing through the tables even without looking at the text. (This is also just a suggestion)
