# Supplementary material for: Flooding and elevated prenatal depression in rural Bangladesh: A mixed methods study
Source: PLOS Glob Public Health. 2025 Jul 21;5(7):e0004792. doi: 10.1371/journal.pgph.0004792 (PMC12279153; doi:10.1371/journal.pgph.0004792)
Supplement: Attachment — Submitted filename: Responses to comments.docx [file pgph.0004792.s010.docx]

We appreciate the thoughtful comments from the Editor and Referees. We have addressed all of their comments, and we feel that the revised manuscript is stronger as a result.

**Academic editor comments**

**1. The introduction provides an overall summary but needs strengthening to focus more on flooding and mental health-related events. As bushfires, earthquakes, and other natural disasters can more severely hamper health service delivery, the authors should focus on the literature on flooding’s impact on the health system and health outcomes. Women/families who experience flooding may also be more income-vulnerable and have other vulnerabilities that increase their baseline risk of prenatal depression; please contextualize that for the study setting of Bangladesh.**

Response: We removed content related to hazards other than flooding and further elaborated on the literature on flooding impacts on the health system. In the third paragraph in the introduction, we revised as follows to highlight the impact of flooding on health systems and health outcomes:

*“Flooding in LMICs could influence prenatal mental health through multiple pathways including reduced agricultural productivity; lower income; damage to household infrastructure; loss of possessions; power outages; lack of access to safe water, sanitation, and hygiene; displacement; trauma; death of family members; disrupted communication systems; and interruptions to prenatal or obstetric care and other health systems.”*

We also added the following paragraph in the Introduction to provide context specific to Bangladesh:

*“Bangladesh ranks as the world’s 7th most climate-affected country (22). Its unique hydrogeology places the majority of the population at flood risk: 75% of the country is within 10 meters above sea level a low-lying, and floodplains constitute 80% of its land mass (14). The seasonal monsoon results in extreme precipitation and frequent flooding in Bangladesh. Forty-five percent of the total population of Bangladesh is at risk of riverine flooding, which is the highest in the world (23), and climate change is predicted to increase the frequency of floods in the future (3). The majority of the population living in floodplains is rural and poor (24). Approximately 4% of the population lives on riverine islands known as chars, where flooding and erosion are even more common. Eighty percent of char residents are ultra-poor, and most do not own land, lack access to healthcare and education, and are excluded from state services. A nationally representative survey of adults from 2019-2020 in Bangladesh found that flooding in the prior year was associated with a 31% higher odds of depression (25). Another study in northeastern Bangladesh on the spatial distribution of mental health outcomes in adults after riverine flooding, found participants with moderate major depressive symptoms lived adjacent to the river (26). However, no prior studies in climate-sensitive populations in Bangladesh have investigated the influence of flooding on prenatal depression.”*

**2. The links between flooding mental health and climate change aren’t clearly and coherently presented; please work on the reviewer's comments to clarify the rationale and provide a stronger synthesis of the literature to frame the study. Please clearly distinguish between floods and natural disasters as a generic term.**

Response: We have revised the introduction to focus specifically on flooding rather than climate change or natural disasters more broadly. We mention climate change to highlight projections for increases in flooding and to discuss global goals related to adaptation. We also revised the following paragraph to strengthen the link between flooding and mental health among pregnant women.

*“Pregnant women face higher risks of mental health conditions including depression and self-harm. Experiencing flooding during pregnancy can result in peritraumatic distress – distress experienced within the period following a traumatic event – and has been shown to increase the risk of prenatal depression in high income settings (7,8). However, no prior studies have investigated the impacts of flooding on prenatal mental health in LMICs, (5) where 26% of women experience perinatal depression (6). Flooding in LMICs could influence prenatal mental health through multiple pathways including reduced agricultural productivity; lower income; damage to household infrastructure; loss of possessions; power outages; lack of access to safe water, sanitation, and hygiene; displacement; trauma; death of family members; disrupted communication systems; and interruptions to prenatal or obstetric care and other health system (4,14–16). In some populations, gender norms require women to stay within the home unless accompanied by men and to keep their clothes dry can make the process of coping with floods more difficult for women, and their typical responsibility to serve as children’s primary caregivers can impede their efforts to adequately prepare for and remain safe during floods (17).”*

**3. Provide added context for the study site (Sirajganj and Tangail districts) and how/why they were selected. Overall, the methods should also provide a research framework that explains why a mixed-method study was considered suitable for the study aims. Additionally, provide details of the randomisation process in the original trial and comment on how the randomisation was done. Ideally, using a difference in difference method to compare intervention (exposure areas) with comparable control areas would have provided more insightful results.**

Response: We added the following details about the study site and the randomization in the first paragraph of the methods section (new text in **bold**):

*“****This study site was chosen because it has a higher prevalence of soil-transmitted helminth infections (the trial’s primary outcome) compared to other regions of Bangladesh (21).*** *The recruitment period for the trial was between August 8, 2023 and July 17, 2024.* ***The trial randomized study households to intervention or control with a one-to-one ratio within geographic blocks of 10 contiguous households.****”*

We revised the beginning of the Methods section to include a justification for our mixed methods design as follows:

*“We used a mixed method approach, triangulating findings from a quantitative cross-sectional baseline survey from a randomized trial and focus group discussions. The quantitative survey was conducted in a large sample of 881 pregnant women, which allowed us to estimate associations between flooding, surface water proximity, and water levels and prenatal depression. The survey also characterized adaptation practices. To further elicit potential mechanisms through which flooding could contribute to prenatal depression, we conducted focus group discussions (FGDs). This approach allowed us to characterize flood-related vulnerabilities and adaptation strategies through in-depth, nuanced discussions and personal narratives that may not be captured in a standardized survey.”*

While we agree with the Editor that the difference-in-difference design is substantially more rigorous than a cross-sectional survey, difference-in-difference designs require data from multiple time points. Currently, only the baseline survey has been completed, so we are not able to conduct this analysis at this time, but in future years we could do so as the trial collects more data.

**4. Please revise Table S1 to give a descriptive analysis of the exposed versus unexposed and comment on their comparability**

Response: We revised this table and the text referring to it as follows:

*“Overall, characteristics were similar between participants who had vs. had not exposed to flooding, however it appeared that women with no flooding exposure had higher socioeconomic status compared to those who had experienced flooding (S1 Table). Mean years of education was higher among those unexposed vs. exposed (6.3 vs. 5.6); the percentage with access to basic sanitation was also slightly higher among those unexposed vs. exposed (17.6% vs. 14.1%).”*

**Reviewer #1:**

**The introduction effectively sets the stage for the study by emphasizing the growing concern of climate change and its impact on mental health, particularly in low- and middle-income countries (LMICs). It provides a strong rationale for focusing on prenatal depression, given its long-term adverse effects on child health and development.
The literature review is thorough, citing relevant studies on the mental health impacts of flooding and the lack of research on prenatal depression in LMICs. The introduction also highlights the unique vulnerabilities of pregnant women in flood-prone areas, such as gender norms and inadequate sanitation, which are critical to understanding the study's context. The introduction could have further emphasized the novelty of the study, particularly its focus on a climate-sensitive community in rural Bangladesh, which is a region highly vulnerable to climate change.**

Response: We added the following paragraph in the Introduction on flooding in Bangladesh to more clearly emphasize the unique focus on this region in this study.

*“Bangladesh ranks as the world’s 7th most climate-affected country (22). Its unique hydrogeology places the majority of the population at flood risk: 75% of the country is within 10 meters above sea level a low-lying, and floodplains constitute 80% of its land mass (14). The seasonal monsoon results in extreme precipitation and frequent flooding in Bangladesh. Forty-five percent of the total population of Bangladesh is at risk of riverine flooding, which is the highest in the world (23), and climate change is predicted to increase the frequency of floods in the future (3). The majority of the population living in floodplains is rural and poor (24). Approximately 4% of the population lives on riverine islands known as chars, where flooding and erosion are even more common. Eighty percent of char residents are ultra-poor, and most do not own land, lack access to healthcare and education, and are excluded from state services. A nationally representative survey of adults from 2019-2020 in Bangladesh found that flooding in the prior year was associated with a 31% higher odds of depression (25). Another study in northeastern Bangladesh on the spatial distribution of mental health outcomes in adults after riverine flooding found participants with moderate major depressive symptoms lived adjacent to the river (26). However, no prior studies in climate-sensitive populations in Bangladesh have investigated the influence of flooding on prenatal depression.”*

Additionally, in the final paragraph in the Introduction, we emphasize the unique climate-vulnerable population that we focused on in this study:

*“In this mixed methods study, including a cross-sectional survey and focus group discussions, our objective was to investigate the association between flooding and prenatal depression in a climate-vulnerable population residing in chars and riverine communities in rural Bangladesh. Additionally, we sought to understand potential drivers of depression following flooding and to identify household strategies for preparing and responding to floods to inform future adaptation interventions for climate resilience. Our overarching aim was to generate evidence to inform climate adaptation strategies that promote women’s mental health, and in turn, healthy births, and child growth and development.”*

**The study employs a mixed-methods approach, combining a cross-sectional survey with focus group discussions. This design is appropriate for capturing both quantitative associations and qualitative insights into the lived experiences of pregnant women in flood-prone areas. The study population consists of 881 pregnant women from riverine communities in rural Bangladesh, a region highly susceptible to flooding. The inclusion criteria are well-defined, focusing on women in their second or third trimester of pregnancy, which is appropriate given the study's focus on prenatal depression. The use of the Edinburgh Postnatal Depression Scale (EPDS) to measure depressive symptoms is appropriate, as it has been validated in similar settings. The inclusion of both self-reported flooding and objective measures of water levels and proximity to surface water strengthens the study's findings.

The statistical methods, including generalized linear models and log-linear models, are appropriate for analyzing the associations between flooding and depression. The use of E-values to assess potential unmeasured confounding is a strength, as it provides a measure of the robustness of the findings. The qualitative component adds depth to the study by identifying key drivers of depression, such as domestic violence, inadequate sanitation, and food insecurity. The thematic analysis approach is well-suited for capturing the nuanced experiences of women in flood-prone areas.

The results are presented clearly, with tables and figures that effectively summarize the key findings. The study finds strong associations between flooding (both compound and latrine flooding) and elevated prenatal depression, as well as thoughts of self-harm additionally with findings which are supported by both quantitative and qualitative data.**

**The focus group discussions reveal important contextual factors, such as the challenges women face in maintaining hygiene and accessing latrines during floods, which contribute to depression. The qualitative data also highlight the limited preparedness of women for flooding events, despite some adaptation strategies like storing food and home modifications. The results are internally consistent, with both self-reported and objective measures of flooding showing similar associations with depression. This consistency strengthens the validity of the findings.**

1. **The discussion could have further explored the implications of the findings for policy and practice, particularly in terms of integrating mental health support into climate adaptation strategies.**

Response: We revised the third paragraph of the discussion as follows (new text in **bold**):

*“Our findings suggest that the adaptation strategies in use in this population may be insufficient for climate resilience* ***and*** ***that it would be valuable to integrate depression screening and mental health support into climate adaptation strategies****. Screening and mental health support may be implemented during antenatal check-ups at government health facilities or through interventions by non-government organizations (NGOs).”*

2. **Unfortunately, the cross-sectional design limits the ability to establish causality between flooding and prenatal depression and the study relies on self-reported data for some measures, which may be subject to recall bias. In the same vein, the lack of data on pre-pregnancy depression and other potential confounders (e.g., domestic violence, social support) may limit the ability to fully account for all factors influencing depression.**

Response: We agree with these points, and our paragraph on limitations addresses each of them. Relevant portions of this paragraph are in bold below:

*“This study was subject to limitations.* ***Estimated associations between flooding and prenatal depression from the quantitative survey may be subject to residual confounding since we did not have data on potential confounders such as pre-pregnancy depression, other mental health conditions, antenatal care, pregnancy symptoms, domestic violence, or social support. However, our E-value analyses showed that confounders would have had to be very strong to explain away associations with home compound and latrine flooding. Also, self-reported flooding may be subject to recall error or courtesy bias; it is possible that women who were depressed may have been more likely to recall flooding, which would have biased estimates away from the null.*** *However, a strength of our study is that we also included objectively measured exposures (distance to surface water, water level); scientific inferences across exposures were similar for analyses of depression scores. While our primary exposure variable was self-reported flooding in the prior 6 months, households located closer to water bodies may have experienced more floods prior to this recall period; flooding may have had cumulative effects on mental health. If so, it is possible that our estimated associations between flooding in the prior 6 months and current depression were over-estimated. An additional limitation is that we did not collect pregnancy status information from focus group participants, so qualitative findings might not fully generalize to the results of the cross-sectional survey of pregnant women. We also did not directly ask about mental health in the focus group discussions, although most discussions centered around the stressful consequences of flooding. This internal consistency between analyses with different exposures supports the validity of our inferences.”*

**Reviewer #2:**

**1. The author should work on grammar flow, starting with the background of the abstract down to the entire document; to improve readability.**

Response: We have reviewed the entire document and made edits throughout to improve clarity and flow.

**2. The author should consider being consistent with the information they present. For example, paragram 2 of the abstract says "We fit generalized linear and log-linear models", however the use of GLM in the results is not apparent.**

Response: for brevity, we did not repeat the name of the models in the results section. We state in the Methods section that GLM models for binary outcomes are reported as prevalence ratios, and those for continuous outcomes are reported as mean differences.

**3. Considering this is a mixed methods study; the presentation of results for both qualitative and quantitative results leaves much to be desired. No proper triangulation of the qualitative and quantitative results. qualitative results have no narration (no apparent story to tell, no context). Labeling of tables in the results section is unclear and hard to follow.**

Response: We have significantly Results and Discussion to triangulate findings from qualitative and quantitative data. We moved content from Box 1 into the Results text and added more quotes and findings from the focus group discussions. We also revised Table 2 to include findings from both qualitative and quantitative data. We revised the Methods as follows to describe our approach:

*“Mixed methods triangulation*

*We triangulated the results of the quantitative cross-sectional survey and the focus group discussions through integrated analysis (32,33). While the quantitative survey was performed in a large sample and is more representative of the population in the study site, the format of the survey may have not elicited a wide range of flood-related vulnerabilities or adaptation strategies that could elucidate mechanisms for the impact of flooding on depression. On the other hand, focus group discussions can elicit the process through which flooding influences mental health, but its smaller sample size may reduce the generalizability of findings. Because the participants in the quantitative and qualitative aspects of our study did not overlap, we analyzed them separately and then we integrated the results from each, giving them equal weight.”*

We revised the table labels of Table 1 to improve clarity.

**4. Study title implies a link between flooding and climate change. However, no clear link is made in the results to speak to the study topic. No climate change modeling was done. Perhaps the author could more specific to what exactly the results speak to and not quickly generalise. Consider building a link that a reader can follow and easily get the message from the text.**

Response: We consider it beyond the scope of the present analysis to try to model mental health outcomes under climate change, particularly because our dataset was collected from a cross-sectional survey with one time point. To more clearly focus the title on the analysis we did, we revised the title to be “Flooding and elevated prenatal depression in rural Bangladesh: a mixed methods study”.

**5. The study identified several drivers such as domestic violence, food insecurity, etc. However, it does not specify if these drivers mediate the relationship between floods and depression, how are these drivers affecting depression? knowing how these drivers affect depression could be useful for readers that are interested in communing up with interventions. You may need to consider doing some mediation analysis.**

Response: Since we conducted a cross-sectional survey, it was not possible to assess mediation of the effect of flooding by these variables. Additionally, our survey did not include quantitative measures of domestic violence and food insecurity; these topics only arose in the qualitative assessment. This is acknowledged under limitations in the discussion section.

**6. The presentation of the ethical considerations for this study leaves much to be desired. Did you get ethical clearance or weaver from IRB? Please check the Journal guidelines on what needs to be cleared stated for this sections.**

Response: The manuscript includes the following at the end of the Methods section in accordance with the journal’s requirements:

*“This study was approved by the International Centre for Diarrhoeal Disease Research, Bangladesh (icddr,b) Ethical Review Committee (PR-22069) and the Stanford Institutional Review Board (63990).*

*Ethics Statement*

*We obtained written consent from pregnant women who participated in the randomized controlled trial and adult women who participated in the FGDs.”*

**Reviewer #3: Overall impression: insightful and resourceful study that explores an ever more prescient issue not only for Bangladesh but other regions where poverty, poor infrastructure, and flood-related climate change events intersect. I appreciate the availability of all the data and coding in order to replicate the results, and to help other researchers conduct similar studies—supporting collaborative international work is paramount in response to climate change.

My only cause for concern: the findings from the FDGs about domestic violence and social ostracization for having to use someone else’s latrine during flooding events in the home. Purdah—and the geosocial context of a poor region in a Muslim country—is barely mentioned, and I believe this needs to be emphasized more strongly throughout the paper. While the authors meet the publishing criteria #4: “Conclusions are presented in an appropriate fashion and are supported by the data,” I am concerned about the lack of data collection around gender-based violence (GBV) especially given the geosocial context of this study, and the lacking discussion of GBV (if it wasn’t feasible to collect this data). I would strongly encourage the authors to explicitly discuss the realities of GBV and climate change as they relate to this study, including the minor mention of domestic violence hidden in the FGD findings, with more emphasis on the sociocultural influence of purdah and stress related to having to leave the home due to flooding, and the probable unmeasured confounding of GBV on prenatal depression in this study. While the e-value is interesting and informative, if there was a factor that could be strong enough to explain some of the associations between flooding and prenatal depression, I think domestic violence would be a good candidate. I think this added context would greatly improve the impact of the paper, especially for readers outside of this social/cultural reality. I don't think I would be the only reader to wonder about this gap in this discussion about GBV and depression among women, in an otherwise well executed and informative study.**

Response: We added additional results related to gender-based violence (GBV) from the focus group participants in the Results section.

*“All FGD participants reported experiencing domestic violence during floods. Participants stated that violence occurs when there is income loss. Participants from both FGDs reported having violent arguments when they ask their husbands for money to buy food during floods when there is limited income and financial losses.*

*‘It is the month of floods. There is no income in the family. So, when I ask for something he (my husband) starts to argue with me. If I tell him to buy some groceries, he starts to hit me. So, I do not express myself, usually out of fear of violence. Still, sometimes I have to tell him when there is no way.’ (FGD participant, age 30)”*

Our quantitative survey did not measure gender-based violence, as it was not a focus of the CRADLE trial, and including it may have had negative, unexpected repercussions for our primary research focus. We agree that GBV may be an important unmeasured confounder, and we have noted this in the limitations section of the Discussion:

*“Estimated associations between flooding and prenatal depression from the quantitative survey may be subject to residual confounding since we did not have data on potential confounders such as pre-pregnancy depression, other mental health conditions, antenatal care, pregnancy symptoms, domestic violence, or social support*.*”*

We added text to the discussion section acknowledging that norms regarding purdah can impact how women take actions to safely relocate during floods. However, purdah was not directly mentioned in the focus group discussions by women. Instead, women implied being shamed based on their wet clothes.

*“Gender norms, including purdah, which requires women to remain within the compound unless accompanied by a male relative, can also prevent women from taking actions to prepare for or safely relocate during floods.”*

**Reviewer #4: The manuscript presents a crucial and rising issue of climate change which is directly and indirectly related to public health concern. Mental health and its importance is further elaborated in the work. Authors have highlighted the vulnerability in the moments of natural disaster like flood which is further aggravated by the climate change impacts. In my review i have following comments and suggestions to improve the article:

1. In general i request authors to have proof read the document after addressing the review queries appropriately.**

Response: We thoroughly proofread the manuscript and edited it prior to resubmitting it.

**2. It will be better not to use numbers and digits while starting the new paragraph and sentence. Please review and address over the manuscript. The citations are not properly formatted over the manuscript, please review and make them uniform.**

Response: We have revised the sentence that started with numbers to start with words instead.

**Abstract
3. Ambiguous terminology used as compounds flooded or compound flooding.
Conclusion of abstract is not well framed but only presented the findings please rewrite them.**

Response: We replaced “compound flooding” with “flooding of compounds” and “home compound flooding”. We also revised the last sentence of the abstract as follows: “*In summary, in rural Bangladesh, flooding, higher water levels, and proximity to water bodies were associated with prenatal depression, and depression following floods was strongly influenced by inadequate sanitation and hygiene infrastructure.”* **Introduction section
4. Setting of background on the study topic would be better in the preliminary section. Clear indication of the study objectives will be better in the introduction section, though authors have indicated the expected outcome.**

Response: As noted in our response to the Editor above, we have substantially revised the introduction to provide background on flooding and depression. We revised the last paragraph of the Introduction as follows:

*“In this mixed methods study, including a cross-sectional survey and focus group discussions, our objective was to investigate the association between flooding and prenatal depression in a low-income, climate-vulnerable setting in rural Bangladesh and to understand potential drivers of depression following flooding. An additional objective was to identify household strategies for preparing and responding to floods to inform future adaptation interventions for climate resilience.”* **Methodology

5. Who and how were FGD participants selected? Were they prenatal group female? If not how do you present the relevance of selecting females out of the prenatal group in this study and their lived experiences associated sith the prenatal group of women?**

Response: The Methods section describes selection of FGD participants as follows (new addition in **bold**):

*“To understand pathways through which flooding increased the risk of prenatal depression and to identify any further preparatory factors contributing to flooding resilience, we analyzed transcripts of focus group discussions (FGDs) conducted as part of an ongoing study related to climate resilience in rural Bangladesh. Participants were women not part of the CRADLE trial but who resided in Sirajganj and Tangail districts and were exposed to flooding risk. FGDs are a method of collecting qualitative data in a small group of participants, administered by a moderator, with prompts and discussion regarding participants’ lived experiences and perceptions (38,39). Participants were adult (age 18+) females residing in the CRADLE study region. We did not collect information about pregnancy status from participants. Two FGDs were conducted in Bengali with 9 to 11 participants per group on August 13 and 14, 2024, and discussions lasted for up to one hour.”*

We clarified in the Methods that we did not collect information about pregnancy status from focus group participants, and we have added the following to the limitations paragraph in the Discussion:

*“An additional limitation is that we did not collect pregnancy status information from focus group participants, so qualitative findings might not fully generalize to the results of the cross-sectional survey of pregnant women.”* **6. Were there any specific mixed methods study designs followed? might be for triangulation? How does the design supports the strengths of this study as claimed by the authors? Also explain in brief any triangulation conducted if any and not presented in the article. I advise to present the triangulation throughout the study for maintaining the scientific rigor of the claimed results and conclusions from the study.**

Response: We have significantly Results and Discussion to triangulate findings from qualitative and quantitative data. We moved content from Box 1 into the Results text and added more quotes and findings from the focus group discussions. We also revised Table 2 to include findings from both qualitative and quantitative data. We revised the Methods as follows to describe our approach:

*“Mixed methods triangulation*

*We triangulated the results of the quantitative cross-sectional survey and the focus group discussions through integrated analysis (32,33). While the quantitative survey was performed in a large sample and is more representative of the population in the study site, the format of the survey may have not elicited a wide range of flood-related vulnerabilities or adaptation strategies that could elucidate mechanisms for the impact of flooding on depression. On the other hand, focus group discussions can elicit the process through which flooding influences mental health, but its smaller sample size may reduce the generalizability of findings. Because the participants in the quantitative and qualitative aspects of our study did not overlap, we analyzed them separately and then we integrated the results from each, giving them equal weight.”*

**Results
7. The results are well explained and presented but will be better if appropriate triangulation conducted for claiming the findings.**

Response: As noted above, we have significantly Results and Discussion to triangulate findings from qualitative and quantitative data. **8. The CRADLE trial mentions 850 as their samples but in the current study the results are presented from 881 pregnant women. Could you explain.**

Response: We clarified this with the following revision in the Methods:

*“The trial randomized study households to intervention or control with a one-to-one ratio within geographic blocks of 10 contiguous households. A minimum 100 meter buffer zone was enforced between all study households to minimize contamination. The baseline survey was conducted in 881 households; 81 of these were excluded from the main trial during the formation of geographic blocks, but we included them in this analysis to maximize the sample size.”* **9. The FGD seems to have been conducted with women only but another query is how the Box 1 could present FGDs with adult men and women.
Additionally; were there any queries asked during FGDs related to health and physical safety which are directly related to prenatal depression and mental health. If yes; how were the responses triangulated with the quantitative findings on Post Natal Depression and flooding. Please present these triangulations as well.**

Response: The FGDs were conducted with women only, we have fixed the error in the title. Women were asked about health problems, challenges and gendered difficulties faced during floods but not about mental health directly. We have included the FGD guidelines in the appendix in the revised manuscript (Appendix item 7). We also revised the Methods as follows to clarify this:

*“Women were asked about health problems, challenges during floods, and gendered difficulties faced during floods but not about mental health directly.”* **Discussions
10. The presentation is well along the risk, vulnerabilities associated with climate change and natural disasters among different locations of Bangladesh. These are well discussed with the concurrent findings from national and international evidences but the chief issue of study association of compound flooding and prenatal depression seems behind. So, it should be further discussed with appropriate triangulations over the section.**

Response: We have substantially revised the discussion section to triangulate results from the quantitative survey and FGDs. Some relevant excerpts are as follows:

*“Our quantitative and qualitative analyses both pointed to inadequate sanitation and hygiene during floods as a potentially important driver of prenatal depression. In the quantitative survey, latrine flooding was associated with 3.6-fold higher moderate or severe depression and 4.3-fold higher severe depression. In FGDs, women mentioned multiple vulnerabilities related to sanitation and hygiene but did not report any adaptation strategies in either the quantitative survey or FGDs (Box 1). During floods, women faced many barriers to bathing, maintaining menstrual hygiene, or relieving themselves; we expect that these particular vulnerabilities would be exacerbated during pregnancy and could be key drivers of prenatal depression.”*

*“FGDs also revealed other potential pathways through which home compound flooding influenced women’s mental health, including displacement, food insecurity, threats to physical safety, children’s risk of drowning, gender-based violence, additional responsibilities in the home, and economic hardship, consistent with prior studies (44).”*

*“The adaptation strategies households used to prepare for and respond to floods were similar to those reported in other studies in Bangladesh. These included temporary relocation to a shelter, raised platform, or boat; raising their home or reinforcing it; storing food; and saving cash. Notably, participants of both the quantitative survey and FGDs mentioned multiple adaptation strategies related to altering their homes during flooding events to reduce the chance of displacement due to damage to their homes or belongings. However, none mentioned preparatory upgrades to their water, sanitation, or hygiene infrastructure.”*

**11. Another query is: Were there any queries during FGDs related to health and safety? How were the responses triangulated with the quantitative findings of flooding associated with prenatal depression.**

Response: Above, we discuss the revisions we made to triangulate results from the quantitative and qualitative data. As noted above, women were asked about health problems, challenges and gendered difficulties faced during floods but not about mental health directly. We have included the FGD guidelines in the appendix in the revised manuscript (Appendix item 7). We revised the Discussion to mention the following limitations:

*“An additional limitation is that we did not collect pregnancy status information from focus group participants, so qualitative findings might not fully generalize to the results of the cross-sectional survey of pregnant women. We also did not directly ask about mental health in the focus group discussions, although most discussions centered around the stressful consequences of flooding.”*

**Reviewer #5: Referee report on “Flooding and elevated prenatal depression in a climate-sensitive community in rural Bangladesh: a mixed methods study” (PGPH-D-25-00092)

The paper aims to examine the associational impact of floods on the mental health outcomes, as measured by depression, amongst pregnant women in rural Bangladesh. In principle, the paper uses a simple linear regression model to examine the likelihood of prenatal depression amongst those women who witnessed a flood incident in the past 6 months in comparison to those who did not. The author(s) report elevated depression levels for the exposed women. The effect are particularly higher if the women experienced latrine flooding. The paper highlights an important issue relating to prenatal depression and the author(s) efforts are appreciable. I have a few suggestions for the author(s) to consider.


Major Comments

1. Empirical Strategy:

(a) While authors state that they are using generalized linear model with Gaussian distribution, as per my understanding, this in essence boils down to a simple linear regression model. I urge the author(s) to remove such redundancy and provide more clarity in this context.**

Response: GLM encompasses a set of models for outcome variables with different distributions, including both linear regression for continuous outcomes and logistic regression for binary outcomes. Because our analysis includes both types of outcomes, we have left the language as is since GLM is the correct overarching term.  **Relatedly, author(s) may consider explicitly writing the regression equation in interest of readers.**

Response: We appreciate the suggestion to include regression equations. However, given the multiple outcomes and models described in this section, including all equations would significantly increase the length of the methods without necessarily improving clarity for most readers. The current description provides a concise yet comprehensive overview of our analytical approach, detailing the outcomes, model types, and adjustment strategy. **(b) The empirical strategy compares difference in outcomes of women witnessing a flood event with the women those did not witness such an event in the last 6 months. While flood is a natural disaster (implying it is exogeneous) -- however there are reasons why flooding may an endogenous regressor.

The reason is that the regions (unions) who witnessed a flood in last 6 months may actually be more likely to witness a flood in comparison to regions which did not witness a flood. This is evident in Figure 2b and 2c. Households that are nearer to seasonal water streams are more likely to report compound (and, perhaps latrine) flooding. If the fact that the regions (unions) who witnessed a flood in last 6 months may actually be more likely to witness a flood in comparison to regions which did not witness a flood, then there is a strong selection bias. In other words, the affected (treated) mothers are more likely to be facing such floods for a continuously more period of time. Thus there remains a possibility that the estimates which the empirical strategy provides may actually be over estimates.

The reason is that floods may induce a perpetual/continued depression -- that may continue beyond 6 months (which is the survey recall horizon). If such affected women (in regions more likely to witness floods) have been suffering from depression for long, then the estimates provided in the table actually represent combining impact of regular flooding rather than floods in the past 6 months.**

**Relatedly, there may be confounders such as:
o Pre-existing mental health conditions:
o Women who already suffer from anxiety or depression might perceive and report flooding as more traumatic.**

Response: Thank you for bringing up this important point. We agree, and we have added the following to the paragraph on limitations in the Discussion:

*“Estimated associations between flooding and prenatal depression from the quantitative survey may be subject to residual confounding since we did not have data on potential confounders such as pre-pregnancy depression, other mental health conditions, antenatal care, pregnancy symptoms, domestic violence, or social support.”*

*“While our primary exposure variable was self-reported flooding in the prior 6 months, households located closer to water bodies may have experienced more floods prior to this recall period; flooding may have had cumulative effects on mental health. If so, it is possible that our estimated associations between flooding in the prior 6 months and current depression were over-estimated.”* **(c) Since authors can potentially know the date when the flood actually occurred (using their data), author(s) can see whether the length of exposure to flood also affects the extent of depression. For instance, are those who suffered a flood just one month prior to the survey more/less likely to be depressed than those who suffered 3 months ago. Related to (b), this may add more insights about the temporal impacts of floods.**

Response: We have added an analysis of duration of latrine flooding with our outcomes in Table S2. We did not find any meaningful associations with depression prevalence or mean EPDS score. While we did collect data on the time elapsed since flooding, the sample size was too small to fit models for time since compound flooding among households with compound flooding (N=32). There were 90 households that reported union flooding, but we were not able to fit adjusted models due to a high level of collinearity between covariates and time since union flooding within this subset of 90 households. We consider this analysis to be less important since we did not find an overall association between depression and union flooding.  **(d) The self-reported flooding data may be prone to recall bias, yet there is no discussion of how this could impact the results.**

Response: We mention this issue in the paragraph on limitations in the Discussion:

*“Also, self-reported flooding may be subject to recall error or courtesy bias; it is possible that women who were depressed may have been more likely to recall flooding, which would have biased estimates away from the null. However, a strength of our study is that we also included objectively measured exposures (distance to surface water, water level); scientific inferences across exposures were similar for analyses of depression scores.”* **(e) Clustering of standard errors: Since the outcomes within regions (unions) may be highly correlated, author(s) may want to cluster their standard errors at the regio(union) level.**

Response: We refit models using Huber-White sandwich standard errors to account for clustering at the block level. We chose the block level instead of the union level because the original randomized trial enrolled households in blocks of 10 geographically contiguous households, so this choice aligns the analysis with the design. We revised the methods as follows and updated results: *“We calculated robust sandwich standard errors to account for clustering within geographic block.”* Please note that the standard errors were larger for continuous outcomes but smaller for binary outcomes, which used Poisson models. Indeed, our original analysis should have used Sandwich standard errors for the Poisson models, for which default standard errors are overly conservative (Zou 2004, *AJE,* DOI: 10.1093/aje/kwh090).

**2. Introduction Section
The author(s) may consider adding two additional paragraphs in the Introduction section. One paragraph for adding the key results, and another additional paragraph for adding the highlighting the key contribution of the paper.**

Response: Our understanding is that for this journal, results are not presented in the Introduction section (we defer to the Editor on whether this change is needed). We revised the final paragraph of the introduction as follows to highlight the contribution of the paper:

*“Our overarching aim was to generate evidence to inform climate adaptation strategies that promote women’s mental health, and in turn, healthy births, and child growth and development.”*

We also added a paragraph on the high level of flood risk faced by populations in Bangladesh, which makes our study population unique given that it is highly climate sensitive:

*“Bangladesh ranks as the world’s 7th most climate-affected country (22). Its unique hydrogeology places the majority of the population at flood risk: 75% of the country is within 10 meters above sea level a low-lying, and floodplains constitute 80% of its land mass (14). The seasonal monsoon results in extreme precipitation and frequent flooding in Bangladesh. Forty-five percent of the total population of Bangladesh is at risk of riverine flooding, which is the highest in the world (23), and climate change is predicted to increase the frequency of floods in the future (3). The majority of the population living in floodplains is rural and poor (24). Approximately 4% of the population lives on riverine islands known as chars, where flooding and erosion are even more common. Eighty percent of char residents are ultra-poor, and most do not own land, lack access to healthcare and education, and are excluded from state services. A nationally representative survey of adults from 2019-2020 in Bangladesh found that flooding in the prior year was associated with a 31% higher odds of depression (25). Another study in northeastern Bangladesh on the spatial distribution of mental health outcomes in adults after riverine flooding found participants with moderate major depressive symptoms lived adjacent to the river (26). However, no prior studies in climate-sensitive populations in Bangladesh have investigated the influence of flooding on prenatal depression.*

**3. Descriptive Statistics
Author(s) may consider separating the descriptive statistics from the main results. A separate section for descriptive statistics may be added.
Relatedly, Table 1 is a little confusing. While I understand N=32, N=3, N=11 (etc) represents certain subsamples. However, their corresponding numbers in column (2) are not percentages. Author(s) may consider revising the table to avoid any source of confusion.**

Response: We added subheaders to the Results section. The first two sections are now “Study participant characteristics” and “Flooding and water exposures”, which present descriptive statistics. Additionally, we revised the heading of Table 1 to clarify that the right-hand column includes percentages or means.

**Minor Comments

1. Table notes
The author(s) may consider providing detailed table notes describing in detail the outcome variables, the independent variable, and the controls along with a brief about methodology (if deemed necessary). The idea is that the reader should be able to understand the paper just by glancing through the tables even without looking at the text. (This is also just a suggestion)**

Response: We have added detailed captions to tables presenting results of regression models.
